# Improving the Use of Social Contact Studies in Epidemic Modeling

**DOI:** 10.1097/EDE.0000000000001876

**Published:** 2025-06-13

**Authors:** Tom Britton, Frank Ball

**Affiliations:** From the a Department of Mathematics, Stockholm University, Stockholm, Sweden; b School of Mathematical Sciences, University of Nottingham, Nottingham, UK.

**Keywords:** Assortativity, Basic reproduction number, Multitype epidemic model, Social contact studies

## Abstract

Social contact studies are used in infectious disease epidemiology to infer a contact matrix M, having the mean number of contacts between individuals of different age groups as elements. However, M does not capture the (often large) variation in the number of contacts within each age group, information is also available in social contact studies. Here, we include such variation by separating each age group into two halves: the socially active (having many contacts) and the socially less active (having fewer contacts). The extended contact matrix and its associated epidemic model show that acknowledging variation in social activity within age groups has a substantial impact on the basic reproduction number, $R_{0}$, and the final fraction getting infected if the epidemic takes off, τ. In fact, variation in social activity is more important for data fitting than allowing for different age groups. A difficulty with variation in social activity, however, is that social contact studies typically lack information on whether mixing with respect to social activity is assortative (when socially active mainly have contact with other socially active individuals) or not. Our analysis shows that accounting for variation in social activity improves model predictability, yielding more accurate expressions for $R_{0}$ and τ irrespective of whether such mixing is assortative, but different assumptions on assortativity give rather different outputs. Future social contact studies should, therefore, also try to infer the degree of assortativity (with respect to social activity) between peers and their contacts.

Research on epidemic models over the last 40 years has made them increasingly realistic by introducing various forms of heterogeneities between individuals. Such modeling extensions only contribute to the analysis of real-world epidemics if they are fitted to data. One such important contribution of heterogeneities has been the use of social contact studies in order to incorporate contact heterogeneities into epidemic models. This began with an important study, analyzing and comparing contact patterns in eight different countries in Europe,^1^ and has continued with many social contact studies in different countries and settings (many such data sources are stored in a previous study^2^).

A social contact study involves a random sample of individuals. After collecting a few individual covariates (age, gender, and household information), researchers ask the sampled individuals to record each close contact (often defined as being within arm-length to an individual for at least one minute) during a 24-hour period, recording their age and other details. The population is divided into age groups i=1,…,k (k=7 age groups is common), and the social contact study is summarized by the age-dependent mean contact matrix $M=\left[\alpha_{ij}\right]$, where element $\alpha_{ij}$ (i and j reflecting two arbitrary age groups) denotes the mean number of contacts that one individual in age group i has with individuals in age group j. Nearly all such empirical contact matrices M show two important features: (1) that older age groups tend to have fewer contacts in total than younger age groups and (2) that all age groups tend to have most contacts with individuals of the same (or similar) age group; the latter feature is referred to as assortative mixing with respect to age.^3^

The matrix M is then incorporated into a multitype epidemic model taking into account that individuals in different age groups have different preferences with which age groups they mix with and also that different age groups have different overall number of contacts (rather than assuming homogeneous mixing). Such multitype epidemic models have been shown to give a better statistical fit to incidence data from epidemic outbreaks (e.g., the study by Wallinga et al.^4^) as compared with the simpler epidemic model neglecting difference between age groups.

The separation of the community into different age groups makes individuals of the same age group resemble each other more with respect to contact patterns. However, there still remains heterogeneity in contact patterns within each age group, and the more heterogeneity (i.e., variation in number of contacts) the cruder is the approximation that assumes that all individuals of the same age group mix similarly. It is worth pointing out that a social contact study carries information at the individual level, so it is easy to study how much variation in social contact remains within each age group. For most social contact studies the answer is “a lot,” that is, more than can be explained by random variations (see the Discussion section for more on this comparison) and we show that acknowledging this extra variation can have an appreciable impact on the predictions of an epidemic model.

In the present paper, we define a new multitype epidemic model that tries to capture also this remaining heterogeneity after separating individuals into different age groups, with the goal to give better model predictability and more accurate expressions for epidemic parameters such as the basic reproduction number $R_{0}$. We do this by dividing each age group into two halves: those with a low overall number of contacts and those with a higher number of contacts. Each age group i is hence divided into two groups, (i,L) and (i,H), for low and high individuals in age group i. For this new group classification, we know how many contacts on average an (i,L) individual has with j individuals, but we do not know what fraction of those contacts are with (j,L) individuals and what fraction are with (j,H) individuals. Since there is no information about these mixing preferences in a social contact study, we consider three different possibilities: the fully assortative case where (i,H) individuals exclusively prioritize (j,H) individuals (and (i,L) individuals exclusively prioritize (j,L) individuals), the intermediate case known as proportionate mixing (with respect to social activity, i.e., number of contacts) in which contacts are selected in the same way for socially active and socially less active individuals, and finally the fully disassortative case where (i,L) individuals only have contact with (j,H) individuals.

Additionally, we consider a simpler epidemic model in which we neglect age completely and instead only divide the population according to how many contacts individuals have in total: the 50% with highest number of social contacts and the 50% with lowest. For this stratification, reflecting social activity only, we derive a matrix of mean number of contacts between the two groups. However, also in this simpler model, data lack information on how the total number of contacts of an individual is divided into its contacts with high-active and low-active individuals. As above we hence consider cases: the fully assortative, proportionate mixing, and the fully disassortative cases.

## METHODS

In the eAppendix; https://links.lww.com/EDE/C251, we give an in-depth description of how social contact study data are used, how they are incorporated into the multitype epidemic model, and properties of a multitype epidemic model; here, we give only a brief outline.

### Social Contact Studies Data

As described above, we make use of empirical datasets from social contact studies. More precisely we have chosen to analyze three social contact studies for which the data are publicly available at https://www.socialcontactdata.org/data/. We chose three datasets representing contacts in Belgium during 2010,^5^ in France during 2012,^6^ and in Vietnam during 2007,^7^ the latter being chosen to explore differences between high-income and low-income countries. More information about each social contact study can be found in the respective references. All datasets were intentionally chosen from before the coronavirus disease 2019 (COVID-19) pandemic, since studies from the pandemic often focused on changes over time as an effect of prevention, something that is not considered here. We emphasize that social contact studies carry information about whom individuals have contact with, they are not connected to any epidemic outbreak. The information is instead used to better mimic contact patterns in epidemic models, patterns that clearly affect how an infectious disease may spread in the community.

Each of the three social contact studies (the studies by Willem et al.,^5^ Béraud et al.,^6^ and Horby et al.^7^) contains information about the sampled individuals and the contacts they have during a given day. The data contain more information, but in the present analyses we use only the ages of the sampled individuals and the ages of their contacts during the day in question. For a given age group i, the contact matrix element $\alpha_{ij}$ is given by the mean number of contacts that sampled i individuals have with individuals in age group j. We adjust these quantities such that the number of contacts of one group to another matches the number of contacts in the opposite direction; without such a correction analyses may lead to misleading conclusions for initial epidemic growth and peak incidence as shown by Hamilton et al.^8^ These adjusted quantities define the contact matrix $M=\left(\alpha_{ij}\right)$ used in the multitype epidemic model taking heterogeneity with respect to age into account (A model).

### Incorporating Social Contact Data into an Epidemic Model

Before defining a multitype epidemic model, we describe how we categorize individuals into different types of individual in four different ways. In each study, we analyze the properties of the homogeneous epidemic model with no heterogeneous structure (Hom), the multitype epidemic model with heterogeneity with respect to age only (A), the multitype epidemic model with heterogeneity with respect to social activity only (S), and the multitype epidemic model with heterogeneity with respect to both age and social activity within age groups (AS), where “social activity” refers to having many or few overall numbers of contacts. Below we hence analyze four different multitype models: Hom (which actually is single-type), A, S, and AS.

Each social contact study carries information on whether sampled individuals are socially active or not, and also which age groups they have contact with. However, it does not contain information on whether the contacted individuals were socially active or not. This is not a problem for the Hom and A models, which do not consider social activity, but for the other two models (S and AS), we overcome this problem by considering three possibilities: assortative mixing, in which socially active people only have contact with other socially active people, and socially less active people only have contact with other socially less active people; disassortative mixing, in which socially active people (mainly) mix with socially less activity people; and the intermediate case of proportionate mixing, in which socially active and socially less active people choose their contacts in the same way.

For the epidemic model taking social activity into account but not age (S model), we divide the sampled individuals into two halves: the 50% having most overall number of contacts and the remaining 50% have fewer overall number of contacts, and the model hence consider two types of individuals. The mean number of contacts in the group with high social activity $m_{H}$ is then computed and similarly $m_{L}$ is computed for the group having low social activity. As discussed in the previous paragraph, social contact studies lack information about whether a contacted individual has high or low social activity, so this we have to hypothesize about. If we assume full assortativity with respect to social activity, then high individuals only have contact with other high individuals, and low individuals only with other low individuals. The contact matrix under this assumption hence has elements $\alpha_{HH}=m_{H}$, $\alpha_{LL}=m_{L}$, and $\alpha_{HL}=\alpha_{LH}=0$.

The fully disassortative model assumes that all contacts from low individuals are with high individuals, but it is not possible for all contacts from high individuals to be with low individuals—there are simply not enough contacts made by low individuals, so the remaining contacts have to be with the high group. Consequently, the disassortative model has the following elements in its contact matrix: $\alpha_{HH}=m_{H}-m_{L}$, $\alpha_{HL}=\alpha_{LH}=m_{L}$, and $\alpha_{LL}=0$.

Finally, in the proportionate mixing assumption (of the S model), each contact, irrespective of whether it comes from a high or low individual, has the probability $p_{H}=m_{H}/\left(m_{H}+m_{L}\right)$ to be with a high individual, and the remaining probability $p_{L}=m_{L}/\left(m_{H}+m_{L}\right)$ to be with a low individual. Hence, the contact matrix M assuming proportionate mixing has elements $\alpha_{HH}=m_{H}p_{H}$, $\alpha_{HL}=m_{H}p_{L}$, $\alpha_{LH}=m_{L}p_{H}$, and $\alpha_{LL}=m_{H}p_{H}$. We have thus defined the 2 × 2 contact matrices M in the S model under the three different mixing assumptions: assortative, disassortative, and proportionate mixing. These are used in the multitype epidemic model described in the next subsection.

In the model taking heterogeneity with respect to both age and social activity into account (AS model), we divide individuals of each age group i into two: (i,H) and (i,L) being the 50% with highest overall social activity and the 50% with lowest overall social activity. It is known how many contacts (i,H) individuals have with individuals in age group j on average (and similarly for (i,L) individuals), but just like in the S model it is not known if these contacts are primarily with (j,H) or (j,L) individuals. Hence, also here we make three different assumptions: assortative mixing, proportionate mixing, and disassortative mixing, all with respect to social activity. The details are given in Section 2 of the eAppendix; https://links.lww.com/EDE/C251, but each gives a contact matrix M of dimension (2 × 7) (2 × 7) = (14 × 14) when there are seven different age groups.

### Multitype Epidemic Model

For each of the three social contact studies, we analyze the four multitype settings Hom, A (7 types), S (2 types), and AS (14 types). For the S and AS models, we consider the fully assortative, the fully disassortative, and the intermediate proportionate-mixing case (the Hom and A models are unaffected, by not considering social activity, and have identical output for the three versions). For each such combination, the respective social contact study gives a mean contact matrix M. Given a specific social contact study and a specific multitype setting, we consider the corresponding stochastic multitype epidemic model. For more details on multitype epidemic models, we refer to Section 4 of the eAppendix; https://links.lww.com/EDE/C251 or Chapter 6 in the study by Andersson and Britton^9^; here, we just give a brief outline. The model assumes that individuals are at first susceptible. If they get infected they first become exposed (latent) and later become infectious. After some time in the infectious state they recover and become immune. Models of this kind are accordingly called susceptible, exposed, infectious, recovered epidemic models.

The multitype susceptible, exposed, infectious, recovered epidemic model (containing the contact matrix M) is defined as follows. individuals are categorized into r different types (our models have r= 1, 7, 2, and 14 types, respectively). We let $\pi_{i}$ denote the community fraction type i (these are available from country-specific demographic data). The basic model assumption is that an individual of type i that gets infected, will on average have $\alpha_{ij}$ contacts with j individuals per day, where $M=\left[\alpha_{ij}\right]$ is the contact matrix described above and inferred from the social contact study. Let $\mu_{I}$ denote the mean duration of the infectious period and p denote the probability that an infectious contact results in infection (assuming the contacted individual is still susceptible), called the transmissibility of the disease in question. The mean number of such infectious contacts an i individual has with j individuals hence equals $\alpha_{ij}\mu_{I}p$. Without loss of generality, we assume that $\mu_{I}=1$ (if not we can redefine the contact matrix M in units of $\mu_{I}$, rather than per day, by multiplying all elements by $\mu_{I}$). Consequently, the transmission probability p is the only remaining free parameter, which is varied in our analyses.

The epidemic has its course, starting with one (or a few) infectious individuals. As the epidemic progresses more people get infected, so more of the infectious contacts are with already infected people, and eventually the epidemic stops.

This multitype epidemic model has been analyzed extensively in the literature, see references in the eAppendix; https://links.lww.com/EDE/C251 or Chapter 6 in the study by Andersson and Britton^9^. It is known that the basic reproduction number $R_{0}$ is given by the largest eigenvalue of pM. If $R_{0}>1$, the epidemic may take off and this happens with probability ρ given by a complicated expression. Further, if the epidemic takes off, then the (random) final fractions getting infected of the different types converge to a deterministic limit as the population size n tends to infinity. These limiting fractions $\tau_{1},\ldots ,\tau_{k}$ are given by the unique strictly positive solution to the k equations


$$\begin{array}{l} 1-\tau_{j}=e^{-p\sum_{i}^{k}\pi_{i}\tau_{i}\alpha_{ij}/\pi_{j}},j=1,\ldots ,k. \end{array}$$

For a given social contact study and multitype setting, we use these methods for computing the basic reproduction number $R_{0}$, the limiting probability of a major outbreak ρ, and the final outbreak size (limiting final fraction getting infected) $\tau =\pi_{1}\tau_{1}+\ldots +\pi_{k}\tau_{k}$, as a function of the overall transmissibility (measured by the transmission probability p).

## RESULTS

**FIGURE 1.**
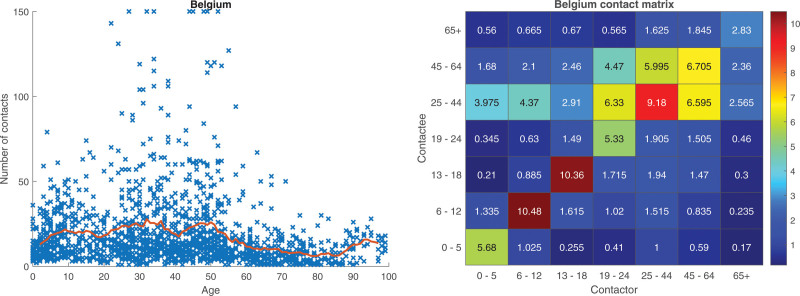
Belgian data.^5^ Scatter plot of the total number of contacts of individuals as a function of age and 4-year moving average as red curve; eight observations with larger values are truncated and set to 150 (left panel). Heatmap representing the contact matrix M between different age groups (right panel).

We start by analyzing the Belgian social contact study^5^ consisting of 1744 individuals. In Figure 1, we plot the total number of contacts (eight observations are truncated at 150 for better visibility) for all individuals as a function of age, and also a heatmap of the contact matrix M. From the scatter plot (left) it is seen that there is large variation in the number of contacts, but also that there is an age effect with the highest overall number of contacts coming from young adults (as seen from the moving average curve). In the heatmap matrix (right), we see strong assortativity (with respect to age) in that individuals mix the most with individuals of similar age.

We next analyze several different epidemic models using the Belgian data, fixing all parameters except the overall transmissibility p, and compute $R_{0}$ and the final outbreak size τ under different model assumptions concerning social contacts.

**FIGURE 2.**
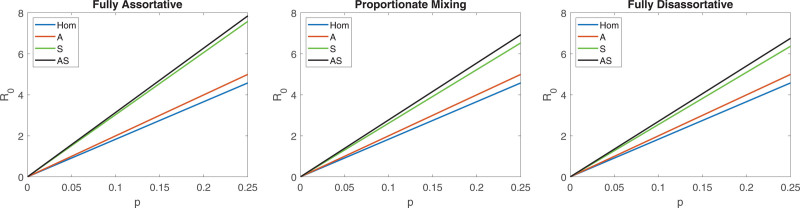
Plot of the basic reproduction number $R_{0}$, as a function of the per contact transmission probability p, for the Belgian contact study,^5^ analyzed using epidemic models acknowledging no heterogeneity (Hom), heterogeneity with respect to age only (A), heterogeneity with respect to social activity only (S), and heterogeneity with respect to both age and social activity (AS), and considering mixing with respect to social activity to be fully assortative (left panel), proportionate mixing (center panel) and fully disassortative right (panel).

In Figure 2, we plot $R_{0}$ as a function of the transmission probability p, for the different models: Hom, A, S, and AS. The left panel shows $R_{0}$ assuming assortativity (with respect to social activity groups), the middle panel assumes proportionate mixing, and the right panel assumes maximal disassortativity. Note that the Hom and the A models do not contain social activity groups, and hence assumptions about mixing between them: their curves are identical in the three panels in all figures and for all datasets. It is the S and AS models that have three versions: assortative, proportionate mixing, and disassortative. It is seen in Figure 2 that $R_{0}$ is linear in the transmissibility p in all models. In each panel, (i) the AS model gives the highest $R_{0}$ and the Hom model gives the lowest $R_{0}$ and (ii) the S model is closest to the AS model and the A model is closest to the Hom model. Further, it is seen that assuming assortative mixing with respect to social activity (left panel) gives the highest $R_{0}$ and the disassortative assumption (right panel) gives the lowest $R_{0}$.

**FIGURE 3.**
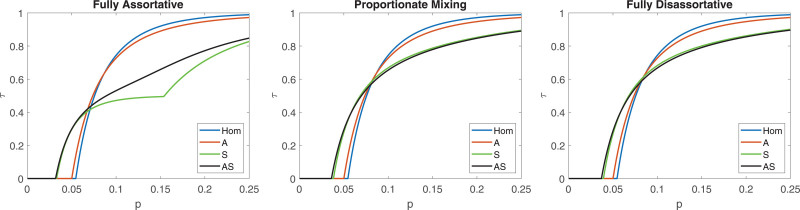
Plot of the final size τ, as a function of the per contact transmission probability p, for the Belgian contact study,^5^ analyzed using epidemic models acknowledging no heterogeneity (Hom), heterogeneity with respect to age only (A), heterogeneity with respect to social activity only (S), and heterogeneity with respect to both age and social activity (AS), and considering mixing with respect to social activity to be fully assortative (left panel), proportionate mixing (center panel) and fully disassortative right (panel).

Next, we plot the final size τ as a function of p for the different models: Hom, A, S, and AS in Figure 3. Similar to above, the left panel assumes complete assortativity, the middle panel proportionate mixing, and the right panel disassortativity. It is seen in each panel, that the A model (taking age into account) resembles the Hom model (assuming no heterogeneities), whereas the S model (heterogeneity with regards to social activity but not age) is fairly similar to the full AS model allowing both types of heterogeneity. None of the four models is of course “true,” but it is reasonable to believe that the AS model lies closest to reality in that it allows for heterogeneities with respect to both age and social activity. Including heterogeneity owing to social activity (S) is, hence, more important than acknowledging heterogeneity with respect to age (A). Further, the observation that the A model lies close to the homogeneous model and the AS model lies close to the S model is a clear indication that social activity classification is more important than allowing for different age groups.

Another observation is that, in each panel, the Hom and A models give smaller outbreaks (compared with the S and AS models) for low overall transmissibility p, whereas the opposite holds true when p is large. An intuitive explanation to this is that strong heterogeneity (S and AS) “helps” an epidemic to take off when p is small, but that when p is large strong heterogeneity “helps” some individuals to escape infection.

If instead we compare the different panels in Figure 3, the final sizes for the S and AS models are different in the three panels (the Hom and A models are identical in the three panels since they do not include social activity). In particular, the left assortative panel is different from the others: the final size is smaller when p is large, and the S model has a type of “bump” at around p=0.15, which is an artifact from having two social activity groups. It is not obvious which of the three panels, assortative, proportionate mixing or disassortative, is the most reasonable but empirical studies, for example, the study by Zhou et al.^10^ indicates that mixing with respect to social activity is assortative, albeit not completely, suggesting that somewhere between left and center panel is closest to reality. Finally, for all three panels, the S model is much closer to the full AS model compared with the A model. Similar conclusions to those from Figure 3 for the final size τ apply also for the major outbreak probability ρ (see eFigure 1 in Section 5.1 of the eAppendix; https://links.lww.com/EDE/C251). As a numerical example we assume p=0.1 and full assortativity. Most modelers would use the A model and conclude that $R_{0}=2.00$ and that the epidemic would result in an outbreak infecting the fraction τ=72.7%, whereas if the better AS model is used the conclusion would instead be $R_{0}=3.14$ and τ=53.1% (so a higher $R_{0}$ but smaller final size). The S model comes much closer to the AS model: $R_{0}=3.03$ and τ=47.1%.

Note that the S model lies closer to the AS model than the A model even though we have seven age groups and only two social activity groups, thus allowing for more heterogeneity for age. In the eAppendix; https://links.lww.com/EDE/C251 (eFigures 2–4 in Section 5.1), we show similar plots when dividing also social activity into seven different levels, and then differences between Hom and A on the one side and S and AS on the other, are even more pronounced.

**FIGURE 4.**
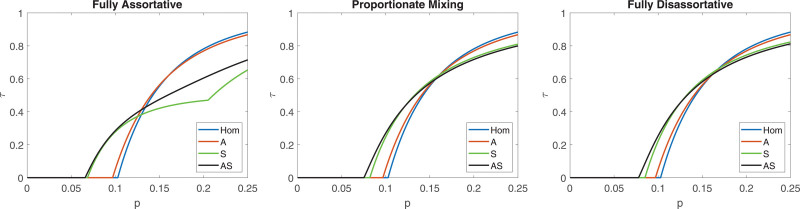
Plot of the final size τ, as a function of the per contact transmission probability p, for the French contact study.^6^ The same panels and assumptions as in Figure 3.

We have done similar comparative analyses for two other social contact studies, which are described more briefly. The conclusions from the French study^6^ are very similar to those from the Belgian analysis, as can be seen in Figure 4 where we plot the final fraction τ getting infected as a function of the overall transmissibility p for the different model assumptions Hom, A, S, and AS, under different assumptions on assortativity with respect to social activity. Additional plots for the French study are given in Section 5.2 of the eAppendix; https://links.lww.com/EDE/C251.

**FIGURE 5.**
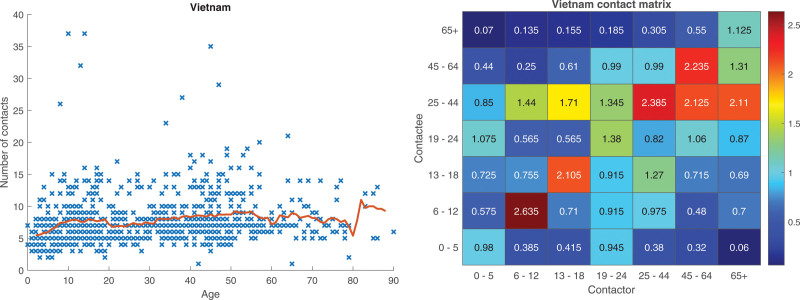
Vietnamese data.^7^ Scatter plot of the total number of contacts of individuals as a function of age, red curve showing the moving average (left panel) and heatmap representing the contact matrix M between different age groups (right panel).

The Vietnamese study^7^ on the other hand shows some differences. The main difference of the Vietnamese social contact study is that the number of contacts varies much less, both within and between age groups, compared to the Belgian and French data. In Figure 5 a scatter plot of the number of contacts is shown as well as a heatmap of the contact matrix with respect to age. It is seen that the variation in the number of contacts is much smaller than in the Belgian data (Figure 1), and that there is still some assortativity for mixing with respect to age with high values on the diagonal.

**FIGURE 6.**
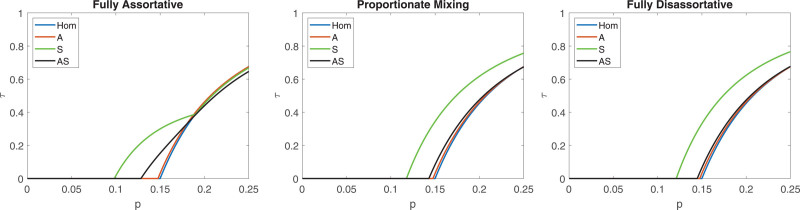
Plot of the final size τ, as a function of the per contact transmission probability p, for the Vietnamese contact study.^7^ The same panels and assumptions as in Figure 3.

When comparing the potential of outbreaks for different models for the Vietnamese study the pattern is different from the Belgian and French studies; see Figure 6. The only model that sticks out now is the S model. Since there is very little variation both within and between age groups, the homogeneous model (Hom) might suffice. The reason why the S model sticks out is probably owing to social activity being the only material source of variability in these data. Additional plots for the Vietnamese study are given in Section 5.3 of the eAppendix; https://links.lww.com/EDE/C251.

## DISCUSSION

The two main insights from our analyses are that (1) heterogeneity in social activity, which is contained in social contact studies, is more important than heterogeneity owing to age when determining what will happen in an epidemic outbreak (traditional use of social contact studies neglects the former and considers only the latter), and (2) whether mixing is assortative with respect to social activity is also an important factor for determining epidemic features such as $R_{0}$ and the final size τ, but current social contact studies lack information on the degree of such assortativity. The first insight implies that epidemic models using social contact studies should include also heterogeneity in social activity within age groups to improve analyses, for example, determining $R_{0}$ and predicting the outbreak size τ. The second insight calls for attention in future social contact studies to investigate also to what degree social mixing is assortative with respect to social activity.

The conclusions are based on three social contact studies from Belgium, France, and Vietnam, and the impact of modeling heterogeneity in social activity within age groups was smallest in the Vietnamese study where variation in the number of contacts was the smallest (the study by Kucharski et al.^11^ report a similar conclusion for a social contact study in Hong Kong). To allow for heterogeneity within age groups is, hence, most important when variation in contacts is large also within age groups. The paradigm in social mixing seems to be that social activity is highly heterogeneous (super-spreaders, scale-free social networks, etc.), so we believe it is the rule rather than the exception that social activity is highly variable also within age groups.

Earlier modeling using social contact studies made use of the contact matrix M having the mean number of contacts between different age cohorts as its elements. But even if there were no systematic differences (in number of contacts) between individuals of the same age group, a social contact study would of course not have all individual contacts of a certain age group being identical—there would clearly be some variation. Therefore, a relevant question is whether the observed variation in contacts within age groups is systematic or more noise-related. We have two strong empirical indicators that variation in contacts really is systematic. The first is that the number of contacts is strongly over-dispersed also within age groups. The variance of the number of contacts divided by the mean number of contacts within each age group varies between 11.4 and 54.3 for the Belgian data and between 13.6 and 26.6 for the French data (but only between 3.7 and 6.8 for the Vietnamese data). If there were no systematic differences between individuals a Poisson distribution seems plausible and then the ratio would lie around 1. The second strong indicator that there are systematic differences in number of contacts also within age groups comes from the French data, where 278 individuals in the study were measured on four different days. (The data comprise two waves, with each wave consisting of two successive days.) By analyzing these 278 individuals, it was found that the correlation between the numbers of contacts on the different days was estimated to lie in the range of 0.30–0.52, a strong positive correlation. If the variation was just noise one would expect no correlation. Hence, there are very strong indications of systematic variation in number of contacts within age groups. It is, however, possible that we overestimate the (systematic) heterogeneity within age groups in our analysis since the separation into activity classes based on observed numbers of contacts is affected by both systematic heterogeneities and noise. An important open problem is, hence, how to disentangle these different sources of variation to improve the estimated systematic differences within age groups.

Social contact studies performed during the COVID-19 pandemic, including the CoMix initiative (the studies by Coletti et al.,^12^ Liu et al.,^13^ and more inhttp://www.socialcontactdata.org/data/^2^), studied changes in social activity during the pandemic. The focus was often on changes in the mean number of contacts between different age groups, but it would be worthwhile to investigate if the reduction in contacts came mainly from socially active individuals reducing their contacts (decreasing dispersion) or mainly from socially less active individuals reducing their contacts even further thus increasing dispersion, or a mixture of both. This is an interesting future research question as these scenarios have different impacts on the potential for disease spreading.

Our modeling focuses on the use of social contact studies and hence lacks several other relevant features in epidemic modeling, for example, seasonality, the effect of local structures such as households and workplaces, and immunity waning. However, we believe that our qualitative conclusions remain valid when adding such features.

## ACKNOWLEDGMENTS

We thank Sue Ball for her assistance with processing the data.

## Supplementary Material
