# Supplementary material for: Improving the Use of Social Contact Studies in Epidemic Modeling
Source: Epidemiology. 2025 Jun 13;36(5):660–7. doi: 10.1097/EDE.0000000000001876 (PMC12303244; doi:10.1097/EDE.0000000000001876)
Supplement: Supplementary file 1 [file ede-36-660-s001.pdf]

Improving the use of social contact studies in epidemic modelling: Supplementary Material

Tom Britton[1]* and Frank Ball[2]

[1]Department of Mathematics, Stockholm University, Sweden
[2]School of Mathematical Sciences, University of Nottingham, UK.

April 28, 2025

1 Contact survey data

The three contact survey data sets we use record details of contacts made by respondents in a fixed period, 1 day for the Belgian and Vietnamese surveys and 2 days for the French survey. For each respondent, we know their age and details of the contacts that they made during the fixed period. In particular, for each contact, information concerning the age of the contacted individual is given, often in the form of an age range $a - b$, where a and b are integers, though sometimes an exact age (in years) is provided. Other information is available, for example concerning the type of contact, but we ignore that in our analysis and only consider the age of contacted individuals. The French data is comprised of two waves. In each wave, participants were observed for 2 consecutive days. Some participants were observed in both waves. In that case we used only their contacts in the first wave in our analysis.

For each contact, if the age of the contacted individual is given as a range, say $a-b$, then the age of the contacted individual was chosen from the discrete uniform distribution on $\{a, a+1, \ldots, b\}$, which we denote by $U(\{a,b\})$. If the age and age range of a contact are both missing then the age of the contacted individual is chosen by sampling uniformly from all contacts made by the respondent, in which either an exact age or an age range, $a - b$ say, is given. If the former, then the contacted individual is assigned that range. If the latter, then the contacted individual is assigned an age chosen from $U(\{a,b\})$. All uniform samplings are independent. In the French analysis, 8 participants were removed as there is no age information for any of their contacts.

[1]Stockholm University, Department of Mathematics, Sweden. E-mail: tom.britton@math.su.se
[2]University of Nottingham, School of Mathematical Sciences, UK.
[3]Stockholm University, Department of Mathematics, Sweden.

For each survey, we split the respondents into 7 age groups, namely $0-5$, $6-12$, $13-18$, $19-24$, $25-44$, $45-64$ and 65+, which we label $1, 2, \dots, 7$. These are the age groups used in the analysis in [5] of the Belgian data, which were based in part on the Belgian schooling system and chosen to reduce sparse data cells in the ensuing contact matrices.

2 Estimation of mean contact matrices

Suppose first that individuals are classified into 7 types based on age: type 1, age $0-5$, type 2, age $6-12$,type 3, age $13-18$, type 4, age $19-24$, type 5, age $25-44$, type 6, age $45-64$ and type 7, age 65+. For $i, j = 1, 2, \dots, 7$, let α_{ij} be the mean number of contacts a given type-i individual has with type-j individuals on a given day. For $i = 1, 2, \dots, 7$, let π_i be the fraction of the population that are of type i. Then the mean total number of contacts type-i individuals have with type-j individuals on a given day, scaled by the total population size is

$$c_{ij} = \pi_i \alpha_{ij}. \tag{1}$$

We obtain an estimate $\hat{\boldsymbol{\pi}}$ of $\boldsymbol{\pi} = (\pi_1, \pi_2, \dots, \pi_7)$ from census data and for each (i, j) we estimate α_{ij} from the contact survey data. Specifically, we estimate α_{ij} by $\hat{\alpha}_{ij}$, the average number of contacts type-i individuals have with type-j individuals in the survey. (For the French data we use half the average number of contacts over the two-day wave.) Note that since a contact is a symmetric event we must have $c_{ij} = c_{ji}$ for all (i, j), so we estimate c_{ij} by

$$\tilde{c}_{ij} = \frac{1}{2}\left(\hat{\pi}_i \hat{\alpha}_{ij} + \hat{\pi}_j \hat{\alpha}_{ji}\right). \tag{2}$$

In more detailed modelling, we also classify individuals according to their activity level. Within an age class, i say, individuals are classified according to the mean total number of contacts (i.e. with individuals of any age group) they make on given day, with those making the top 50% mean number of contacts being type (i, H) and those making the bottom 50% mean number of contacts being type (i, L). For $i, j = 1, 2, \dots, 7$, let α^H_{ij} be the mean number of contacts a given type-(i, H) individual has with type-j individuals on a given day and define α^L_{ij} similarly. The mean total number of contacts (scaled by population size) made with type-j individuals on a given day by type-(i, H) and type-(i, L) individuals are, respectively,

$$c^H_{ij} = \pi_i \alpha^H_{ij} \qquad \text{and} \qquad c^L_{ij} = \pi_i \alpha^L_{ij}.$$

For each (i, j), we obtain estimates $\hat{\alpha}^H_{ij}$ and $\hat{\alpha}^L_{ij}$ of α^H_{ij} and α^L_{ij}, respectively, from the contact survey data as follows. We order the participants in age group i according to the total number of contacts they make. If the number of participants in age group i is even then we split them into two equal-sized groups, a low activity-group and a high-activity group according to the above ordering. Then, $\hat{\alpha}^H_{ij}$ is the mean number of contacts made with type-j individuals by high-activity type-i individuals in the survey and $\hat{\alpha}^L_{ij}$ is defined similarly. If the number of participants in age group i is odd then we proceed similarly, except the middle participant in the ordering is assigned with weight $\frac{1}{2}$ to both groups and $\hat{\alpha}^H_{ij}$ and $\hat{\alpha}^L_{ij}$ are obtained using weighted averages.

The fraction of contacts made by type-i individuals with type-j individuals that emanate from type-(i, H) individuals is

$$\frac{\alpha_{ij}^H}{\alpha_{ij}^H + \alpha_{ij}^L} = \frac{\alpha_{ij}^H}{2\alpha_{ij}},$$

so we estimate c_{ij}^H and c_{ij}^L by

$$\tilde{c}_{ij}^H = \frac{\hat{\alpha}_{ij}^H}{2\hat{\alpha}_{ij}}\tilde{c}_{ij} \qquad \text{and} \qquad \tilde{c}_{ij}^L = \frac{\hat{\alpha}_{ij}^L}{2\hat{\alpha}_{ij}}\tilde{c}_{ij},$$

respectively.

For $i, j = 1, 2, \ldots, 7$, let c_{ij}^{HH} be the mean number of contacts made by type-(i, H) individuals with type-(j, H) individuals on a given day and define c_{ij}^{HL}, c_{ij}^{LH} and c_{ij}^{LL} similarly. We need estimates of these quantities to calculate epidemic model properties but they are not available from the contact data, as the identities of contacted individuals are not given. Thus, we consider the two extreme cases, where mixing is (i) as assortative as possible and (ii) as disassortative as possible, and also proportionate mixing.

In the assortative case, we use the estimates

$$\begin{bmatrix} \tilde{c}_{ij}^{LL,A} & \tilde{c}_{ij}^{LH,A} \\ \tilde{c}_{ij}^{HL,A} & \tilde{c}_{ij}^{HH,A} \end{bmatrix} = \begin{bmatrix} \min(\tilde{c}_{ij}^L, \tilde{c}_{ji}^L) & \tilde{c}_{ij}^L - \min(\tilde{c}_{ij}^L, \tilde{c}_{ji}^L) \\ \tilde{c}_{ij}^H - \min(\tilde{c}_{ij}^H, \tilde{c}_{ji}^H) & \min(\tilde{c}_{ij}^H, \tilde{c}_{ji}^H) \end{bmatrix}. \tag{3}$$

The estimates $\tilde{c}_{ij}^{LL,A}$ and $\tilde{c}_{ij}^{HH,A}$ are the largest possible consistent with symmetry constraints. Note also that, since $\tilde{c}_{ji} = \tilde{c}_{ji}^H + \tilde{c}_{ji}^L$,

$$\begin{aligned} \tilde{c}_{ij}^{HL,A} - \tilde{c}_{ji}^{LH,A} &= \tilde{c}_{ij}^H - \min(\tilde{c}_{ij}^H, \tilde{c}_{ji}^H) - \tilde{c}_{ji}^L + \min(\tilde{c}_{ji}^L, \tilde{c}_{ij}^L) \\ &= \tilde{c}_{ij}^H - \min(\tilde{c}_{ij}^H, \tilde{c}_{ji}^H) - \tilde{c}_{ji} + \tilde{c}_{ji}^H + \min(\tilde{c}_{ji} - \tilde{c}_{ji}^H, \tilde{c}_{ji} - \tilde{c}_{ij}^H) \\ &= \tilde{c}_{ij}^H - \min(\tilde{c}_{ij}^H, \tilde{c}_{ji}^H) + \tilde{c}_{ji}^H - \max(\tilde{c}_{ji}^H, \tilde{c}_{ij}^H) \\ &= 0. \end{aligned}$$

In the disassortative case, we use the estimates

$$\begin{bmatrix} \tilde{c}_{ij}^{LL,D} & \tilde{c}_{ij}^{LH,D} \\ \tilde{c}_{ij}^{HL,D} & \tilde{c}_{ij}^{HH,D} \end{bmatrix} = \begin{bmatrix} 0 & \tilde{c}_{ij}^L \\ \tilde{c}_{ji}^L & \tilde{c}_{ij}^H - \tilde{c}_{ji}^L \end{bmatrix}. \tag{4}$$

The estimates $\tilde{c}_{ij}^{LL,D}, \tilde{c}_{ij}^{LH,D}$ and $\tilde{c}_{ij}^{HL,D}$ clearly yield the greatest disassortativity, while maintaining symmetry. Note that, using $\tilde{c}_{ij} = \tilde{c}_{ji}$,

$$\tilde{c}_{ij}^H - \tilde{c}_{ji}^L = \left(\frac{\hat{\alpha}_{ij}^H}{2\hat{\alpha}_{ij}} - \frac{\hat{\alpha}_{ji}^L}{2\hat{\alpha}_{ji}} \right) \tilde{c}_{ij} \geq 0,$$

since $\frac{\hat{\alpha}_{ij}^H}{2\hat{\alpha}_{ij}} \geq \frac{1}{2}$ and $\frac{\hat{\alpha}_{ji}^L}{2\hat{\alpha}_{ji}} \leq \frac{1}{2}$.

For proportionate mixing, we use the estimates

$$\begin{bmatrix} \tilde{c}_{ij}^{LL,P} & \tilde{c}_{ij}^{LH,P} \\ \tilde{c}_{ij}^{HL,P} & \tilde{c}_{ij}^{HH,P} \end{bmatrix} = \begin{bmatrix} \tilde{c}_{ij}^L \frac{\tilde{c}_{ji}^L}{\tilde{c}_{ji}^L + \tilde{c}_{ji}^H} & \tilde{c}_{ij}^L \frac{\tilde{c}_{ji}^H}{\tilde{c}_{ji}^L + \tilde{c}_{ji}^H} \\ \tilde{c}_{ij}^H \frac{\tilde{c}_{ji}^L}{\tilde{c}_{ji}^L + \tilde{c}_{ji}^H} & \tilde{c}_{ij}^H \frac{\tilde{c}_{ji}^H}{\tilde{c}_{ji}^L + \tilde{c}_{ji}^H} \end{bmatrix}. \tag{5}$$

For example, $\tilde{c}_{ij}^{LL,P}$ and $\tilde{c}_{ij}^{LH,P}$ are obtained by noting that the mean number of contacts made by type-(i,L) individuals with type-j individuals on a given day is $\tilde{c}_{ij}^{L}$, and assuming that these contacts are distributed between type-(j,L) and type-(j,H) individuals in the same proportion as the mean daily numbers of contacts made by type-(j,L) and type-(j,H) individuals with type-i individuals. Since $\tilde{c}_{ij}^{L}+\tilde{c}_{ij}^{H}=\tilde{c}_{ji}^{L}+\tilde{c}_{ji}^{H}$ for all i,j, it is easily checked that the estimates in (5) satisfy the required symmetry constraints.

Consider now the model in which age is neglected and the population is split into d equally-sized groups based on their total number of contacts. In our analysis we use $d=2$ and $d=7$. We label the groups, equivalently type the individuals, $1,2,\dots,d$, in increasing order of activity level. For $i=1,2,\dots,d$, let $\alpha_i^{(d)}$ be the mean total number of contacts made by a given type-i individual in a day. For a given survey, we order all of the respondents according to the total number of contacts they make, split the ordered list into d equally-sized groups and then estimate $\alpha_i^{(d)}$ by $\hat{\alpha}_i^{(d)}$, the mean total number of contacts made by individuals in the ith group. (If the number of respondents is not divisible by d then we make the groups as equally sized as possible.) We consider two extreme models: in which mixing between groups is (i) fully assortative and (ii) as disassoratitive possible, while still satisfying symmetry constraints.

For $i,j=1,2,\dots,d$, let $\alpha_{ij}^{(d,A)}$ be the mean daily total number of contacts made by a given type-i individual with type-j individuals in the assortative case, and define $\alpha_{ij}^{(d,D)}$ and $\alpha_{ij}^{(d,P)}$ similarly for the disassortative and proportionate mixing cases, respectively. We estimate $\alpha_{ij}^{(d,A)}$ by

$$\hat{\alpha}_{ij}^{(d,A)}=\begin{cases}\hat{\alpha}_i^{(d)} & \text{if } i=j,\\ 0 & \text{if } i\neq j.\end{cases} \tag{6}$$

Note that since the population groups are equally sized, $\pi_i^{(d)}=d^{-1}$ $(i=1,2,\dots,d)$, where $\pi_i^{(d)}$ is the fraction of the population that is of type i. Clearly, $\pi_i^{(d)}\hat{\alpha}_{ij}^{(d,A)}=\pi_j^{(d)}\hat{\alpha}_{ji}^{(d,A)}$ for all $i,j=1,2,\dots,d$.

Turning to the diasassortative case, let $\hat{\beta}_0^{(d)}=0$ and for $i=1,2,\dots,d$, let $\hat{\beta}_i^{(d)}=\sum_{j=1}^{i}\hat{\alpha}_j^{(d)}$. For $i=1,2,\dots,d$, let $\hat{F}_i=(\hat{\beta}_{i-1}^{(d)},\hat{\beta}_i^{(d)})$ and $\hat{G}_i=(1-\hat{\beta}_i^{(d)},1-\hat{\beta}_{i-1}^{(d)})$. For $i,j=1,2,\dots,d$, we estimate $\alpha_{ij}^{(d,D)}$ by

$$\hat{\alpha}_{ij}^{(d,D)}=|\hat{F}_i\cap\hat{G}_j|.$$

Note that $\hat{F}_i\cap\hat{G}_j$ is a (possibly empty) interval and $|\hat{F}_i\cap\hat{G}_j|$ is the length of that interval. Again, it is easily checked that $\pi_i^{(d)}\hat{\alpha}_{ij}^{(d,D)}=\pi_j^{(d)}\hat{\alpha}_{ji}^{(d,D)}$ for all $i,j=1,2,\dots,d$.

For proportionate mixing we estimate $\alpha_{ij}^{(d,P)}$ by

$$\hat{\alpha}_{ij}^{(d,P)}=\hat{\alpha}_i^{(d)}\frac{\hat{\alpha}_j^{(d)}}{\sum_{l=1}^{d}\hat{\alpha}_l^{(d)}}\qquad (i,j=1,2,\dots,d).$$

The justification for these estimates is similar to that for the estimates in (5) and it is easily seen that $\pi_i^{(d)}\hat{\alpha}_{ij}^{(d,P)}=\pi_j^{(d)}\hat{\alpha}_{ji}^{(d,P)}$ for all $i,j=1,2,\dots,d$.

Age group i	N_i	$\hat{\pi}_i$
0-5	781.6	0.0702
6-12	864.4	0.0776
13-18	748.7	0.0672
19-24	835.4	0.0750
25-44	2949.2	0.2648
45-64	2999.2	0.2693
65+	1959.5	0.1759

Table 1: Belgian age-cohort frequencies

Age group	Frequency
0–4	8.5
5–9	8.0
10–14	8.5
15–19	10.2
20–24	9.2
25–29	8.9
30–34	7.9
35–39	7.6
40–44	7.0
45–49	6.4
50–54	5.3
55–59	3.6
60–64	2.3
65+ 6.6	5.3

Table 2: Vietnamese age-cohort frequencies from census data

3 Estimation of fractions of the population in the age groups

For analysis of the Belgian contact data, the number of individuals in each cohort (N_i) in thousands were found from demographic data (Eurostat, 2013) and converted into fractions yielding the estimates in Table 1

For analysis of the Vietnamese contact data, we used age-cohort frequencies from a UN census study given in Table 2

The age cohorts in these data are different from those in our analysis. To obtain estimates for the age cohorts in our analysis, we used Table 2 to calculate the fractions of each age 0,1,. . . , 64, assuming uniform spread within each cohort, and then summed these over the cohorts in our analysis to obtain the estimates given in Table 3.

For analysis of the French contact data, the age cohorts in demographic data were taken from January 1, 2014 (even though the study was performed in 2012) since older data is not provided in Eurostat, Data Browser, but we expect very similar results for data two years earlier. This yielded the estimates given in Table 4.

Age group	0-5	6-12	13-18	19-24	25-44	45-64	65+
$\hat{\pi}_i$	0.1010	0.1150	0.1156	0.1124	0.3140	0.1760	0.0660

Table 3: Vietnamese age-cohort fractions used in analysis

Age group	0-5	6-12	13-18	19-24	25-44	45-64	65+
$\hat{\pi}_i$	0.0734	0.0876	0.0738	0.0706	0.2538	0.2612	0.1796

Table 4: French age-cohort fractions used in analysis

4 Multitype SEIR epidemic model and basic properties

We use the multitype SEIR stochastic epidemic model in a closed population. The SEIR epidemic model is an extension of the SIR epidemic model, by allowing for a latent period (E for Exposed) upon infection prior to becoming infectious. The final outcome of an SEIR epidemic model is identical to that of the corresponding SIR epidemic model (without latent periods) – latent periods affect only the time dynamics of such epidemics. For more about final outcome properties of the multitype SEIR epidemic it is hence possible to study properties of the multitype SIR epidemic model as described, for example, in [1], Chapter 6, which should be consulted for further details. There are k types of individuals, labelled $1, 2, \ldots, k$. Thus, $k = 7$ in the age-structured model and $k = 14$ in the model with both age groups and activity levels. Initially there are n_i susceptibles and m_i infectives of type i, $i = 1, 2, \ldots, k$. Let $n = n_1+n_2+\cdots+n_k$ and $m = m_1+m_2+\cdots+m_k$ be the total numbers of initial susceptibles and infectives, respectively. For $i = 1, 2, \ldots, k$, let $\pi_i = n^{-1}n_i$ be the fraction of initial susceptibles that are of type i. The infectious periods of infectives are independent and identically distributed according to an exponential distribution with mean $\mu_I = \gamma^{-1}$. For $i, j = 1, 2, \ldots, k$, during their infectious period a given type-i infective makes infectious contact with a given type-j susceptible at the points of a homogeneous Poisson process having rate $n^{-1}\lambda_{ij}$. We consider the situation where the initial number of susceptibles, n, is large and the initial number of infectives m is small.

Let $\Lambda = [\lambda_{ij}]$, $\boldsymbol{\pi} = (\pi_1, \pi_2, \ldots, \pi_k)$ and $\Pi = \text{diag}(\boldsymbol{\pi})$ be the $k \times k$ diagonal matrix whose consecutive diagonal elements are given by $\boldsymbol{\pi}$. We assume that the epidemic is irreducible, i.e. that $\pi_i > 0$ for all i and there exists an integer $n_0 > 0$ such that every element of Λ^{n_0} is strictly positive. The basic reproduction number R_0 of the epidemic is given by the maximal eigenvalue of the next-generation matrix $\mu_I \Lambda \Pi$.

The probability of a major outbreak is zero if $R_0 \leq 1$. If $R_0 > 1$, then a major outbreak occurs with probability

$$1 - \prod_{i=1}^{k} q_i^{m_i}, \tag{7}$$

where $(q_1, q_2, \ldots, q_k)$ is the unique solution in $[0, 1)^k$ of

$$q_j = \frac{\gamma}{\gamma + \sum_{i=1}^{k} \lambda_{ji}(1 - q_i)\pi_i} \qquad (j = 1, 2, \ldots, k). \tag{8}$$

If the population is fully susceptible and an epidemic is started by an individual chosen uniformly at random from the population being infected then the probability of a major

outbreak is

$$\rho = 1 - \sum_{j=1}^{k} \pi_j q_j. \tag{9}$$

Suppose that $R_0 > 1$ and a major outbreak occurs. For $j = 1, 2, \ldots, k$, let τ_j be the fraction of type-j susceptibles that are ultimately infected by the epidemic. Then, $(\tau_1, \tau_2, \ldots, \tau_k)$ is the unique solution in $(0, 1]^k$ of

$$1 - \tau_j = \exp\left(-\mu_I \sum_{i=1}^{k} \pi_i \tau_i \lambda_{ij}\right) \qquad (j = 1, 2, \ldots, k). \tag{10}$$

The overall fraction of susceptibles that are ultimately infected by the epidemic, τ say, is given by

$$\tau = \sum_{j=1}^{k} \pi_j \tau_j. \tag{11}$$

The expression for R_0 continues to hold if the infectious period follows an arbitrary but specified distribution having mean μ_I, as do the expressions (10) and (11) concerning the final size of a major outbreak. The expressions (7) and (9) concerning the probability of a major outbreak also hold but the equations (8) governing $(q_1, q_2, \ldots, q_k)$ change; see [2] for details. All the quantities considered here (i.e. $R_0, q_1, q_2, \ldots, q_k, \rho$, $\tau_1, \tau_2, \ldots, \tau_k$ and τ) are invariant to the introduction of a latent period into the model.

Suppose that $k = 1$, so the population is homogeneously mixing, and let $\lambda = \lambda_{11}$. Then $R_0 = \frac{\lambda}{\gamma}$ and if $R_0 > 1$, then $\rho = 1 - R_0^{-1}$ and τ is the unique strictly positive solution of $1 - \tau = \exp(-\mu_I \tau)$. (If $R_0 \leq 1$, then $\rho = \tau = 0$.)

4.1 Estimation of epidemic model parameters $(\Lambda, \boldsymbol{\pi})$

Consider the age-structured model. Note that $\lambda_{ij}\pi_j$ is the total rate that a given type-i infective makes infectious contact with type-j individuals. Recall that α_{ij} is the mean number of contacts a given type-i individual has with type-j individuals on a given day. Thus, if the transmission probability given a single contact is p, we have

$$\alpha_{ij} p = \lambda_{ij} \pi_j \qquad (i, j = 1, 2, \ldots, d), \tag{12}$$

and using (1) yields $\lambda_{ij} = p\pi_i^{-1} c_{ij} \pi_j^{-1}$. Hence,

$$\Lambda = p\Pi^{-1} C \Pi^{-1}, \tag{13}$$

where $C = [c_{ij}]$. As mentioned previously, we obtain from census data an estimate $\hat{\boldsymbol{\pi}}$ of $\boldsymbol{\pi}$ and hence an estimate $\hat{\Pi}$ of Π. Using (13), we estimate Λ by

$$\tilde{\Lambda} = p\hat{\Pi}^{-1} \tilde{C} \hat{\Pi}^{-1}, \tag{14}$$

where $\tilde{C} = [\tilde{c}_{ij}]$, with $\tilde{c}_{ij}$ being given by (2).

Recall that in the model with age structure and activity levels the types are (i, L) $(i = 1, 2, \ldots, 7)$ and (i, H) $(i = 1, 2, \ldots, 7)$. As each age group is split into two equally-sized groups, we have in an obvious notation that

$$\pi_{i,L} = \pi_{i,H} = \frac{\pi_i}{2} \qquad (i = 1, 2, \ldots, 7).$$

The 14 types are ordered $(1, L), (1, H), (2, L), (2, H), \ldots, (7, L), (7, H)$ for computing purposes. The diagonal matrix Π now has dimension 14 and its estimate $\hat{\Pi}$ is obtained in the obvious fashion. Let C^A and Λ^A be respectively the (14×14) contact and infection rate matrices in the assortative case and define (C^D, Λ^D) and (C^P, Λ^P) similarly for the disassortative and proportionate mixing cases, respectively. The matrix C^A can be partitioned into 7×7 blocks of 2×2 matrices, each of which may be estimated using (3), yielding an estimate, $\tilde{C}^A$ say, of C^A. We then estimate Λ^A by $\tilde{\Lambda}^A$, obtained by replacing $\tilde{C}$ by $\tilde{C}^A$ in (14). The estimates $\tilde{\Lambda}^D$ of Λ^D and $\tilde{\Lambda}^P$ of Λ^P are obtained similarly, using estimates of $\tilde{C}^D$ of C^D and $\tilde{C}^P$ of C^P based on (4) and (5), respectively.

For the model in which age is neglected and the population is split into d equally-sized groups based on individuals' total number of contacts, $\pi_i^{(d)} = d^{-1}$ $(i = 1, 2, \ldots, d)$ and an analogous equation to (12) yields that in an obvious notation

$$\hat{\lambda}_{ij}^{(d,A)} = dp\hat{\alpha}_{ij}^{(d,A)}, \quad \hat{\lambda}_{ij}^{(d,D)} = dp\hat{\alpha}_{ij}^{(d,D)} \quad \text{and} \quad \hat{\lambda}_{ij}^{(d,P)} = dp\hat{\alpha}_{ij}^{(d,P)} \qquad (i, j = 1, 2, \ldots, d).$$

Note that $\hat{\lambda}_{ij}^{(d,A)} = 0$ unless $i = j$, so the epidemic is not irreducible and the above theory cannot be applied directly. In this case, there is no interaction between the d activity groups, so an epidemic with a single initial infective spreads only in the group of that infective. Each of the d activity groups has its own R_0, ρ and τ, denoted respectively by $R_0^{(i)}, \rho^{(i)}$ and $\tau^{(i)}$ for group i, which can be calculated using the $k = 1$ results at the end of Section 4. These $R_0^{(i)}$, $\rho^{(i)}$ and $\tau^{(i)}$ are each strictly increasing in i as the groups are labelled in increasing order of activity. In the figures showing the basic reproduction number of this model, $R_0 = R_0^{(d)} = \max\{R_0^{(i)} : i = 1, 2, \ldots, d\}$ is plotted. The plots of the major outbreak probability ρ show

$$\rho = \sum_{i=1}^{d} \pi_i^{(d)} \rho^{(i)} = d^{-1} \sum_{i=1}^{d} \rho^{(i)}. \tag{15}$$

Thus, ρ is the probability that an epidemic initiated by an individual chosen uniformly at random from the population takes off within that individual's group. The plots of the final size τ show

$$\tau = \sum_{i=1}^{d} \pi_i^{(d)} \tau^{(i)} = d^{-1} \sum_{i=1}^{d} \tau^{(i)}. \tag{16}$$

Note that this τ is not the fraction of the whole community that is infected by a major outbreak, which cannot exceed $d^{-1}\tau^{(d)}$. Instead, τ is the limiting fraction of the community infected by a major outbreak if a small fraction ϵ of contacts are with individuals of a different type and $\epsilon \downarrow 0$, for example, if (6) is replaced by

$$\hat{\alpha}_{ij}^{(d,A)} = \begin{cases} (1-\epsilon)\hat{\alpha}_i^{(d)} & \text{if } i = j, \\ (d-1)^{-1}\epsilon\hat{\alpha}_i^{(d)} & \text{if } i \neq j. \end{cases}$$

As the transmission probability p increases from 0, a new non-zero term enters the sums in (15) and (16) when an $R_0^{(i)}(p)$ crosses the threshold one, which explains the kinks in the plots of ρ and τ for this model.

Finally, for the homogeneously mixing model, note that the mean total number of contacts made by a typical individual on a given day is $\sum_{i=1}^{7} \pi_i \sum_{j=1}^{7} \alpha_{ij}$, so we estimate λ by $\hat{\lambda} = \sum_{i=1}^{7} \hat{\pi}_i \sum_{j=1}^{7} \hat{\alpha}_{ij}$.

5 Additional illustrations

5.1 The Belgian Social Contact study

In Figure 1 we plot the limiting probability of a major outbreak ρ as a function of p for the Belgian contact study under the various models.

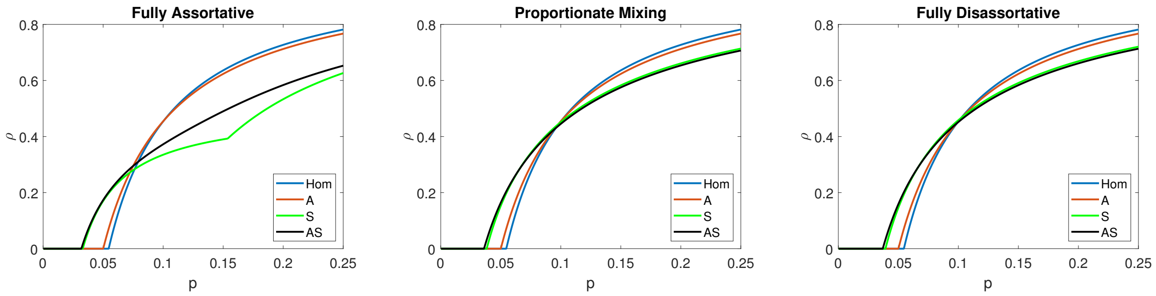

Figure 1: Plot of the major outbreak probability ρ, as a function of the per contact transmission probability p, for the Belgian contact study [5], analysed using epidemic models acknowledging no heterogeneity (Hom), heterogeneity with respect to age only (A), heterogeneity with respect to social activity only (S), and heterogeneity with respect to both age and social activity (AS), and considering mixing with respect to social activity to be fully assortative (left panel), proportionate mixing (center panel) and fully disassortative right (panel).

In Figures 2-4 we show plots of R_0, τ and ρ for the Belgian contact study under the various models, where in the S-model social activity is divided into 7 levels.

5.2 The French Social Contact study

In Figure 5, we show for the French data a scatter plot of the number of contacts made by individuals against age and a heatmap of the corresponding contact matrix.

In Figures 6 and 6, we show plots of R_0 and ρ as a function of p for the French data.

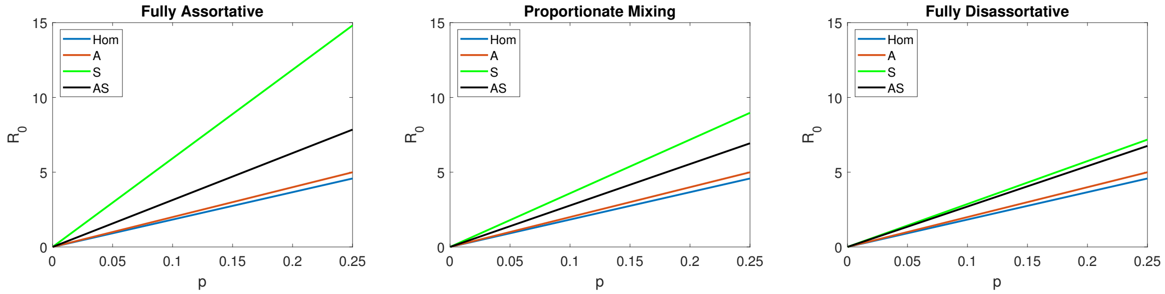

Figure 2: Plot of the basic reproduction number R_0, as a function of the per contact transmission probability p, for the Belgian contact study [5], analysed using epidemic models acknowledging no heterogeneity (Hom), heterogeneity with respect to age only (A), heterogeneity with respect to social activity only split into 7 groups (S), and heterogeneity with respect to both age and social activity (AS), and considering mixing with respect to social activity to be fully assortative (left panel), proportionate mixing (center panel) and fully disassortative right (panel).

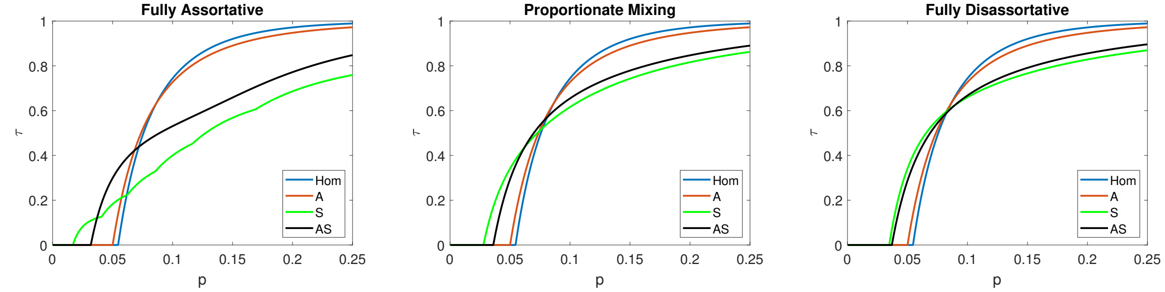

Figure 3: Plot of the final size τ, as a function of the per contact transmission probability p, for the Belgian contact study [5], analysed using epidemic models acknowledging no heterogeneity (No), heterogeneity with respect to age only (A), heterogeneity with respect to social activity only split into 7 groups (S), and heterogeneity with respect to both age and social activity (AS), and considering mixing with respect to social activity to be fully assortative (left panel), proportionate mixing (center panel) and fully disassortative right (panel).

5.3 The Vietnamese Social Contact study

In Figures 8 and 8, we show plots of R_0 and ρ as a function of p for the Viatnamese data.

References

[1] H Andersson and T Britton (2000) Stochastic Epidemic Models and their Statistical Analysis, Springer, New York.

[2] Ball F. and Clancy D. (1993). The final size and severity of a generalised stochastic multitype epidemic model. *Advances in Applied Probability* 25: 721-736.

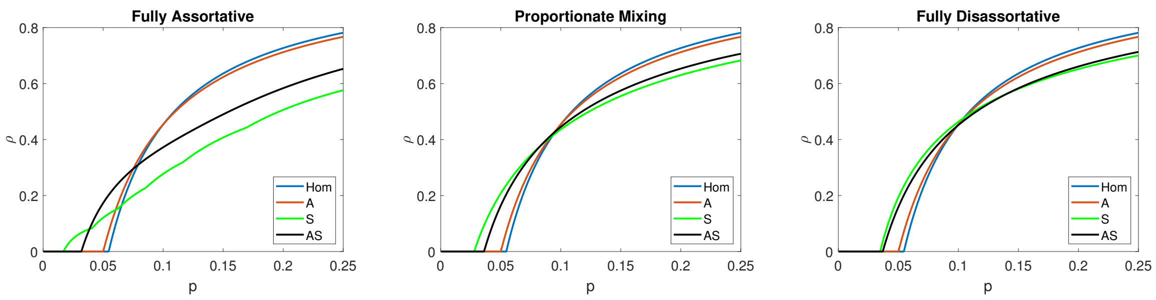

Figure 4: Plot of the major outbreak probability ρ, as a function of the per contact transmission probability p, for the Belgian contact study [5], analysed using epidemic models acknowledging no heterogeneity (Hom), heterogeneity with respect to age only (A), heterogeneity with respect to social activity only split into 7 groups (S), and heterogeneity with respect to both age and social activity (AS), and considering mixing with respect to social activity to be fully assortative (left panel), proportionate mixing (center panel) and fully disassortative right (panel).

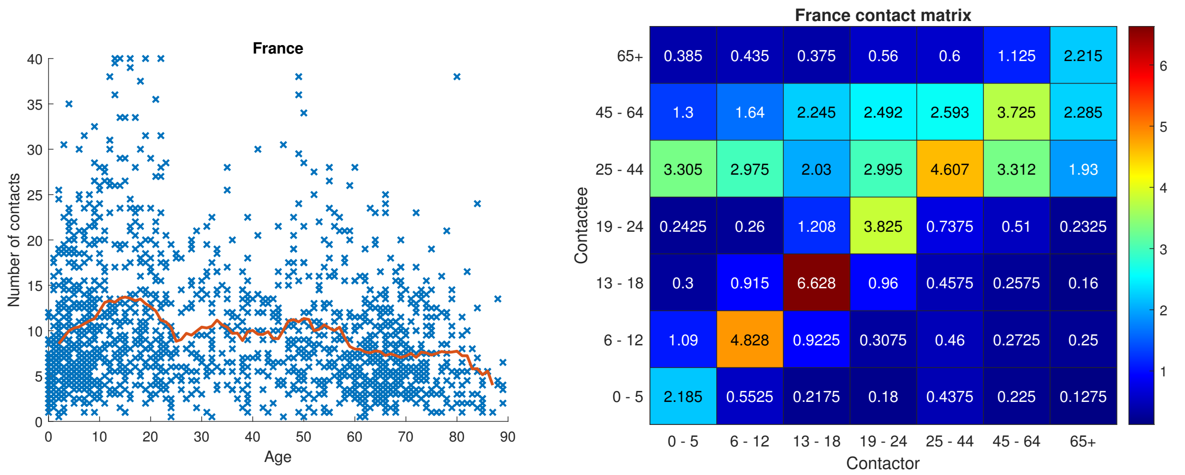

Figure 5: French data [3]. Scatter plot of the total number of contacts of individuals as a function of age (left panel) and heatmap representing the contact matrix C between different age-groups (right panel).

[3] G Béraud, S Kazmercziak, P Beutels, D Levy-Bruhl, X Lenne et al. (2015). The French Connection: The First Large Population-Based Contact Survey in France Relevant for the Spread of Infectious Diseases. *PLoS One*, DOI:10.1371/journal.pone.0133203.

[4] Horby P, Thai PQ, Hens N, Yen NTT, Mai LQ, et al. (2011) Social Contact Patterns in Vietnam and Implications for the Control of Infectious Diseases. *PLoS One* 6(2): e16965. doi:10.1371/journal.pone.0016965

[5] Willem L, Van Kerckhove K, Chao DL, Hens N, Beutels P. (2012). A nice day for an infection? Weather conditions and social contact patterns relevant to influenza transmission. *PloS One* 7(11):e48695.

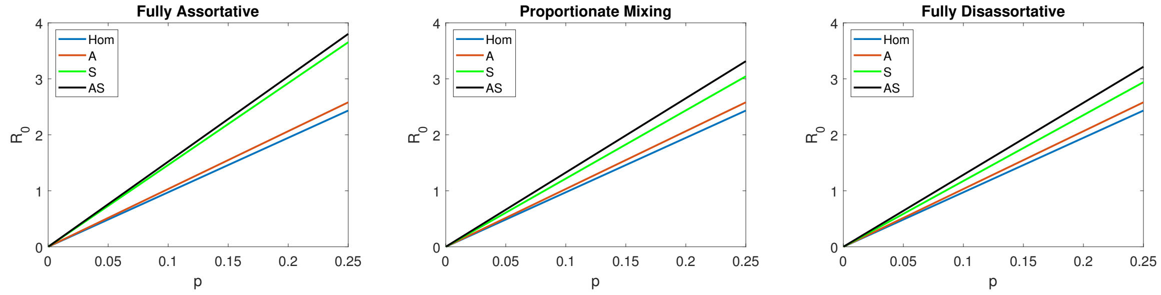

Figure 6: Plot of the basic reproduction number R_0, as a function of the per contact transmission probability p, for the French contact study [3], analysed using epidemic models acknowledging no heterogeneity (Hom), heterogeneity with respect to age only (A), heterogeneity with respect to social activity only (S), and heterogeneity with respect to both age and social activity (AS), and considering mixing with respect to social activity to be fully assortative (left panel), proportionate mixing (center panel) and fully disassortative right (panel).

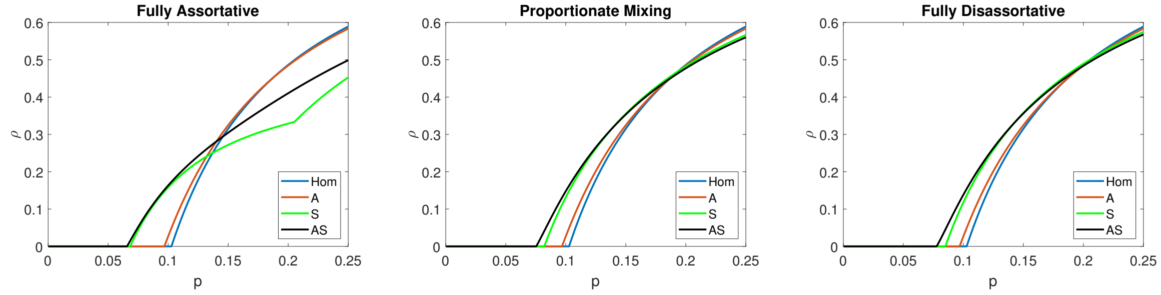

Figure 7: Plot of the major outbreak probability ρ, as a function of the per contact transmission probability p, for the French contact study [3], analysed using epidemic models acknowledging no heterogeneity (Hom), heterogeneity with respect to age only (A), heterogeneity with respect to social activity only (S), and heterogeneity with respect to both age and social activity (AS), and considering mixing with respect to social activity to be fully assortative (left panel), proportionate mixing (center panel) and fully disassortative right (panel).

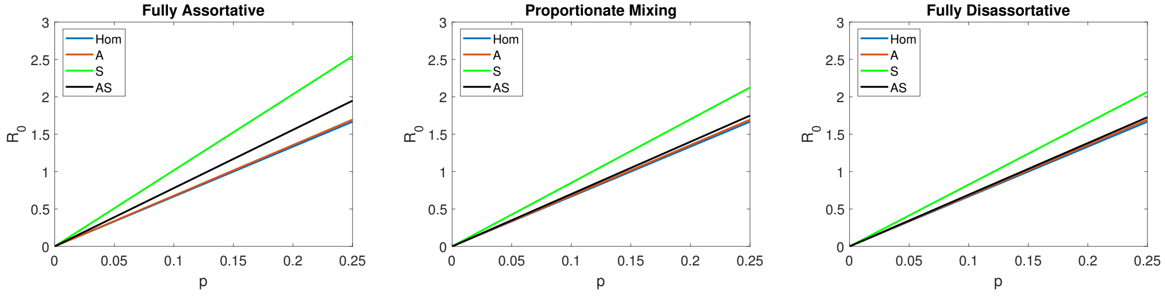

Figure 8: Plot of the basic reproduction number R_0, as a function of the per contact transmission probability p, for the Vietnamese contact study [4], analysed using epidemic models acknowledging no heterogeneity (Hom), heterogeneity with respect to age only (A), heterogeneity with respect to social activity only (S), and heterogeneity with respect to both age and social activity (AS), and considering mixing with respect to social activity to be fully assortative (left panel), proportionate mixing (center panel) and fully disassortative right (panel).

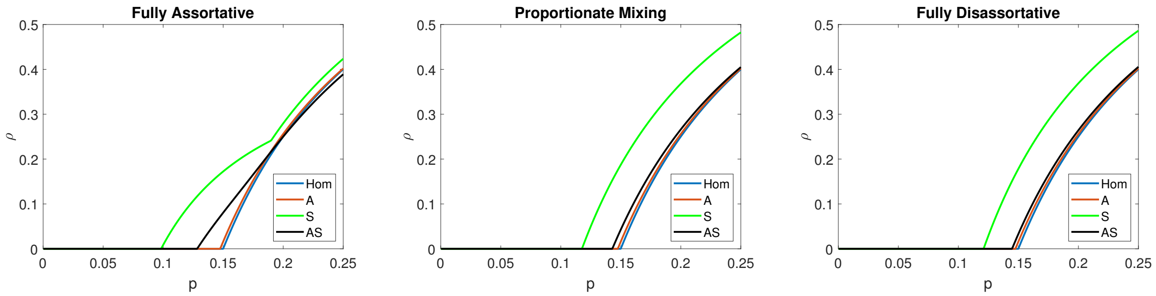

Figure 9: Plot of the major outbreak probability ρ, as a function of the per contact transmission probability p, for the Vietnamese contact study [4], analysed using epidemic models acknowledging no heterogeneity (Hom), heterogeneity with respect to age only (A), heterogeneity with respect to social activity only (S), and heterogeneity with respect to both age and social activity (AS), and considering mixing with respect to social activity to be fully assortative (left panel), proportionate mixing (center panel) and fully disassortative right (panel).
